# Electrospun Poly(ε-caprolactone) Composite Nanofibers with Controlled Release of *Cis*-Diamminediiodoplatinum for a Higher Anticancer Activity

**DOI:** 10.1186/s11671-017-2092-y

**Published:** 2017-04-28

**Authors:** Chaojing Mu, Qingsheng Wu

**Affiliations:** School of Chemical Science and Engineering, Shanghai Key Lab of Chemical Assessment and Sustainability, Tongji University, Shanghai, 200092 China

**Keywords:** Electrospun nanofibers, Drug carrier, *Cis*-diamminediiodoplatinum, Controlled release, Anticancer reagent

## Abstract

Poly(ε-caprolactone) (PCL) nanofibers were prepared by electrospun, on which the *cis*-diamminediiodoplatinum (*cis-*DIDP) was loaded, *cis*-DIDP@PCL, which effectively overcame *cis-*DIDP from dissociation or premature interaction with other bimolecular groups. Meanwhile, the toxicity and cross-resistance of *cis*-DIDP were reduced greatly. In vitro, *cis-*DIDP released from the PCL nanofibers eradicated the tumor cells around twice times more than free *cis-*DIDP, even better than cisplatin. Furthermore, *cis*-DIDP@PCL could controllably release *cis-*DIDP in different sustained-release solution based on our experiment.

**Electronic supplementary material:**

The online version of this article (doi:10.1186/s11671-017-2092-y) contains supplementary material, which is available to authorized users.

## Background

In the 1960s, Rosenberg and colleagues accidentally discovered the cytotoxicity of cisplatin (*cis-*Pt [NH_3_]_2_Cl_2_, Additional file 1: Figure S1a), which showed high anticancer activity [1]. At the end of 1970s, cisplatin became the first platinum anticancer drugs in clinic [2]. Then, it was widely used for the treatment of many malignancies, including testicular, ovarian, bladder, head and neck, small-cell, and non-small-cell lung cancers [3]. Dozens of cisplatin analogs, such as carboplatin, oxaliplatin, nedaplatin, and lobaplatin, were synthesized and used in some limited range [4]. However, the efficacies of cisplatin and its analogs were primarily restricted by their poor water solubility, toxicity, and cross-resistance [5]. The rapid development of nanotechnology had promoted the in-depth study of platinum anticancer drugs [6]. *Cis*-diamminediiodoplatinum (*cis*-DIDP) is now mainly used as the intermediate preparing cisplatin and other analogs [7]. With square planar structure, the *cis*-DIDP is similar to cisplatin, but chlorine ion (Cl^−^) is substituted by iodine ion (I^−^). According to the spectrochemical sequence of crystal field theory, *cis-*DIDP is more unstable than cisplatin. Therefore, in solution, I^−^ is easier to leave than Cl^−^, so I^−^ is more reactive than Cl^−^. In other words, in platinum complexes, I^−^ is more readily being substituted than Cl^−^ by solvent water molecules, which makes it possible for *cis-*DIDP to act as an anticancer reagent with better activity than cisplatin [8]. It is to be expected that *cis-*DIDP could directly act as an efficient anticancer reagent rather than as an intermediate. There might be some methods to improve the therapeutic indices of platinum anticancer drugs, i.e., the development of cancer-targeting formulations of platinum-containing drugs, including drug carriers such as polymer, long-circulating liposome, and polymeric micelle [9–11]. The development of controlled release drug carrier makes it possible for *cis-*DIDP to be applied in clinical.

Electrospinning is a direct and relatively easy method to fabricate ultra-fine fibers with average diameters in the range of sub-micrometer down to nanometer [12, 13]. In this process, continuous polymer liquid strand is drawn through a spinneret needle by a high electrostatic force to deposit randomly on a grounded collector as non-woven fibers. These fibers exhibit interesting characteristics, for example, higher surface area to mass or volume ratio, smaller inter-fibrous pore size with high porosity, and vast possibilities for surface fictionalizations [12, 14]. Due to these advantages, fibers prepared by electrospun have been recently used as new controlled release drug carrier, [15–17] which can lower overall medicinal dosages, improve therapeutic efficacy, reduce toxicity by delivering drugs into the lesion location, and release drug at controlled rate [18]. With good biocompatibility, many polymers were used as medical materials, [19] even used in anticancer drugs [20]. For good drug permeability, poly(ε-caprolactone) (PCL) fibers are now widely used as drug carriers and surgical sutures [21–23]. PCL fibers were selected as the delivery vehicle for some characteristics such as biocompatible, biodegradable characteristic, and PCL could be eliminated from the body dissolved in body fluid without side effect [24, 25].

The first time, we reported that the *cis-*DIDP was loaded on PCL fibers by electrospun to overcome its instability, poor-water-solubility, toxicity, and cross-resistance. The drug loading efficiency of *cis-*DIDP@PCL was assessed; releasing profiles and anticancer activity were tested in vitro. Ultraviolet–visible spectroscopy (UV–Vis) had been handily used to detect the hydrolysis of platinum complexes. Degraded in vitro, the *cis-*DIDP@PCL might be used as a vehicle for anticancer drug to improve cancer chemotherapy both in safety and efficacy. It was interesting to note that *cis-*DIDP can act not only as intermediate to prepare other platinum-based drugs but also as anticancer reagent.

## Methods

### Experimental

#### Chemicals and Materials

Cisplatin and *cis-*DIDP were purchased from *Kunming Guiyan Pharmaceutical Co. Ltd*. (China) and stored away from light at −4 °C. PCL (molecular weight 5 × 10^4^), sodium chlorine (A.R.), glycine (B.R.), and glucose (A.R.) were purchased from *Sinopharm Chemical Reagent Co. Ltd.* (China). RPMI1640 (the culture medium) and newborn calf serum were purchased from *Shanghai Shichen Reagent Co. Ltd.* (China). Human hepatocellular carcinoma cell line SMMC-7721 was newly purchased from *Shanghai Cell Center* (Chinese Academy of Sciences).

#### UV of Cisplatin and *Cis-*DIDP

Cisplatin and *cis-*DIDP were dissolved respectively away from light in deionized water (different solutions such as normal saline, 5% glucose, and 0.1 mol L^−1^ glycine were respectively used as alternative solvent to examine the solvent effect) to form 1 mmol L^−1^ solution. The solution absorbance was determined from time to time by the Agilent 8453 UV–Vis spectrophotometer (*Agilent*, USA).

#### Preparation of *Cis-*DIDP@PCL

PCL was dissolved in dimethylformamide (DMF) to form a polymer solution (PCL wt% 5–15%), and heated by water bath. Then, a predetermined amount of *cis-*DIDP (1–15% to PCL) was dispersed in DMF. The *cis-*DIDP dispersion was added into the PCL polymer solution with continuous stirring to form homogeneous PCL polymer solution containing *cis-*DIDP. In the electrospinning procedure, the polymer solution was firstly transferred to a syringe. Then, the syringe pump was used to deliver the solution through a hollow needle (8#, outside diameter of the needle is 8 mm), the flow rates were 0.5–3.0 mL h^−1^. A high voltage DC generator was used to produce 10–25 kV voltage to inject polymer solution through the hollow needle. An aluminum foil was used as a collector to gather the random fibers. The distances from the spinneret to the collector were 10–25 cm. All the experiments were performed at room temperature. The fibers were finally taken out and dried under vacuum for 48 h. The blank PCL fibers were fabricated by the same method but without dispersing the *cis-*DIDP in the DMF dichloromethane solution.

The fibers with average diameters from 50 to 500 nm could be fine-tuned by adjusting electrospinning parameters, such as concentration of *cis*-DIDP, solvent, electrospinning voltage, polymer solution flow rates, and the distances between needle and collector. Different operation parameters are listed in Additional file 1: Tables S1–S4. Additional file 1: Figures S5–S8 shows the SEM images of the products fabricated under different conditions. After trial and error, the following electrospinning conditions were used: 10/100 (*cis*-DIDP/PCL), 20 kV (voltage), 1.0 mL h^−1^ (flow rate), 15 cm (distance) to prepare products for further studies.

#### Characterizations

The products generally were characterized by SEM, XRD, and FT-IR [26]. An S-4800 high-resolution field-emission scanning electron microscopy (FE-SEM, *Hitachi*, Japan) was used to observe the morphology of collected fibers. The samples for SEM observation were sputtered and coated with a thin layer of gold for better imaging. The average fiber diameters and its distribution were calculated from the random fibers of a typical SEM image.

The structure of *cis-*DIDP powders, PCL, and nanofibers were examined by Advance D8 X-ray diffraction (XRD, *Bruker*, Germany). The XRD patterns were determined with an X-ray diffractometer with Cu Ka radiation (*λ* = 1.54056 Å, 40 kV, 40 mA) over the 2*θ* range of 10°–70° with the scanning rate of 0.2°s^−1^.

FT-IR (*Thermo Fisher*, USA) was used to analyze the molecular structure of *cis*-DIDP, blank PCL nanofibers, and *cis*-DIDP@PCL nanofibers. Drug *cis*-DIDP was commonly mixed with potassium bromide (KBr) and compressed to pellet; nanofibers were cut into pieces and mixed with KBr and compressed to pellets, then were scanned at the wave number of 4000–400 cm^−1^.

#### Release Profile In Vitro and Loading Efficiency

The mass of *cis-*DIDP in solution were determined by UV–Vis spectrophotometer. The release profile of *cis-*DIDP was obtained from *cis-*DIDP@PCL immersion in deionized water, normal saline, or phosphate buffer solution (PBS), respectively. The *cis-*DIDP@PCL (~100 mg each) was statically incubated in 100 mL deionized water, normal saline, or PBS (pH 7.4), as sustained-release solution, respectively. At preset interval, 1 mL incubated solution was taken out and measured by UV–Vis spectrophotometer, and meanwhile, 1 mL solution was added into the sustained-release solution. The experiments were performed for three times, using the immersion solution of blank fibers as control. The accumulative release of *cis-*DIDP from the fiber was calculated as a function of the incubation time. In this paper, *cis-*DIDP was uniformly dispersed in the electrospinning solution and evenly scattered in the PCL fibers [27, 28]. Predetermined amount of *cis-*DIDP@PCL (~100 mg) was dissolved in 100 mL sustained-release solution. The concentration of *cis-*DIDP was measured by UV–Vis spectroscopy for three times. Because of uniform dispersion of *cis-*DIDP in solution and scattered in the fibers, the encapsulation efficiency (%EE) of the product could be calculated by Eq. (1).

1 $$ \%\mathrm{E}\mathrm{E}=\left(1{\textstyle\ \hbox{-}\ }{C}_0\times {V}_0\times {10}^{-3}\right)\times 100\%/\left({M}_0\times \mathrm{Wt}\%\right) $$

Here, *C*_0_ is the concentration of *cis-*DIDP in *cis-*DIDP@PCL (μg mL^−1^), *V*_0_ is the volume of *cis-*DIDP@PCL solution (mL), *M*_0_ is the mass of added *cis-*DIDP@PCL (mg), and wt% is the mass fraction of *cis-*DIDP in fiber.

#### Anticancer Activity In Vitro

In vitro, the anticancer activity of the *cis-*DIDP and *cis-*DIDP@PCL fibers were examined by MTT assay; cisplatin was selected as control. Human hepatocellular carcinoma cells (SMMC-7721 line cell) were chosen as the target tumor cells. The tumor cells were cultured in RPMI 1640 containing 10% newborn calf serum, 25 μg mL^−1^ penicillin and 25 μg mL^−1^ streptomycin, then adjusted to 5 × 10^4^ cells mL^−1^; 200 μL aliquots of the cell suspension were added into each well of a 108-well plate and incubated in the humidified atmosphere containing 5% CO_2_ at 30°C for 24 h. Cisplatin, *cis-*DIDP, and *cis-*DIDP@PCL were added to the tumor-cell-cultured well and incubated for another 12, 24, 36, 48, and 72 h, respectively. Cisplatin, *cis-*DIDP, and *cis-*DIDP in *cis-*DIDP@PCL contents were 50 μg mL^−1^. The 20 μL MTT solution (5 mg mL^−1^) was added to each well and maintained incubation for 4 h. Finally, the supernatant in the wells was discarded carefully, and 150 μL DMSO was added to each of the wells to dissolve the residue. The optical densities of DMSO solutions were determined by a microplate reader at 490 nm, and the cell inhibition was calculated.

## Results and Discussion

### Comparison of *Cis-*DIDP and Cisplatin by UV Irradiation

**Fig. 1:**
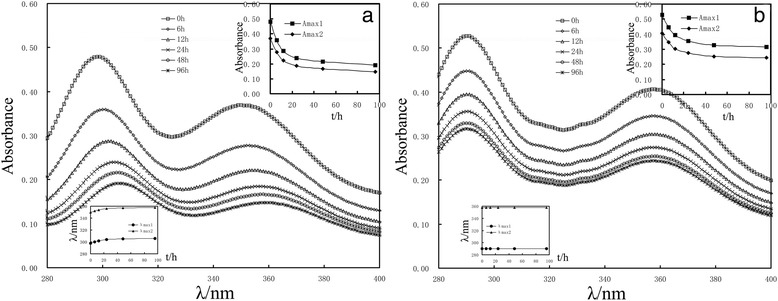
UV absorbance changes with time of 1 mmol L^−1^ *cis*-DIDP (**a**) and cisplatin (**b**) in deionized water. *White square* 0 h, *white diamond* 6 h, *white up-pointing triangle* 12 h, *multiplication sign* 24 h, *white circle* 48 h, *asterisk* 96 h

Figure 1 showed the time-dependent changes of ultraviolet absorbance in deionized water of 1 mmol L^−1^ *cis-*DIDP (Fig. 1a) and cisplatin (Fig. 1b). There were two strong initial UV absorbance peaks of *cis-*DIDP at 298 and 350 nm and that of cisplatin at 290 and 358 nm in deionized water. Comparing cisplatin (seen in the lower left corner in Fig. 1b), the *cis-*DIDP UV absorbance with larger redshift could be seen (seen in the lower left corner in Fig. 1a). These results showed that in deionized water, the UV absorbance of *cis-*DIDP and cisplatin gradually decreased with the time increasing (hypochromic effect, □-0 h, ◇-6 h, △-12 h, ×-24 h, ○-48 h, *-96 h). The same trend could be seen in other aqueous solution (Additional file 1: Figures S2–S4). Compared to that in deionized water, the concentration for both of *cis-*DIDP and cisplatin decreases slower in the normal saline, faster in 5% glucose and fastest in 0.1 mol L^−1^ glycine. The results indicated that the presence of chloride ions inhibits the hydrolysis of *cis-*DIDP and cisplatin; however, the presence of biological molecules accelerated the hydrolysis. Notice the UV absorption peaks of *cis-*DIDP decreased more than that of cisplatin in the aqueous solution (seen the upper right corner in Fig. 1). Accordingly, the *cis-*DIDP was hydrolyzed more rapidly than cisplatin in deionized water.

### Morphology and Structure of the Products

**Fig. 2:**
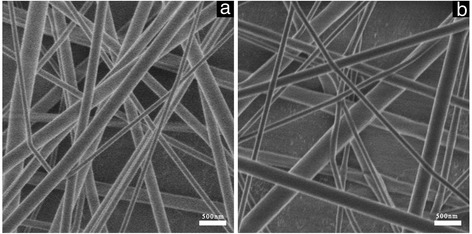
SEM micrograph of the **a** typical blank PCL fibers and **b** *cis*-DIDP@PCL fibers

As shown in Fig. 2, the diameters of PCL nanofibers (Fig. 2a) are 60–350 nm. After loading *cis*-DIDP, the diameters of *cis-*DIDP@PCL (Fig. 2b) reached 100–500 nm. The *cis-*DIDP@PCL appear uniform, and no particles are observed on the smooth PCL nanofiber surface, suggesting that *cis-*DIDP is finely dispersed on the surface of PCL nanofibers or encapsulated into the fiber pores.

**Fig. 3:**
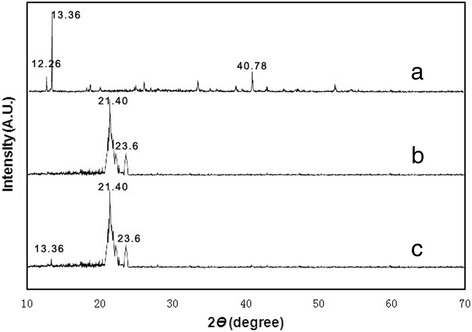
XRD patterns. **a** *Cis*-DIDP powders. **b** PCL fibers. **c** *Cis*-DIDP@PCL fibers

To demonstrate the physical state of *cis-*DIDP in the nanofibers, *cis-*DIDP powders, PCL nanofibers, and *cis-*DIDP@PCL were characterized by XRD. Figure 3 shows the XRD patterns of the *cis-*DIDP powders (Fig. 3a), PCL nanofibers (Fig. 3b), and *cis-*DIDP@PCL (Fig. 3c). The *cis-*DIDP powders are crystalline (Fig. 3a), with characteristic peaks at 2*θ* = 12.26°, 13.36°, 40.78°, while the PCL nanofibers characteristic peaks are at 2θ = 21.40°, 23.60°. As shown in Fig. 3c, very little crystalline *cis-*DIDP was detected in the *cis-*DIDP@PCL, suggesting that *cis-*DIDP was dispersed in the PCL nanofibers uniformly.

**Fig. 4:**
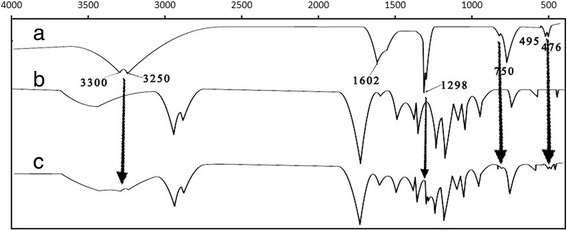
FT-IR curve. **a** *Cis*-DIDP powders. **b** PCL fibers. **c** *Cis*-DIDP@PCL fibers

To demonstrate the feature of *cis*-DIDP combination within PCL nanofibers, the molecular structure of *cis*-DIDP powders, blank PCL nanofibers, and *cis*-DIDP@PCL were analyzed by infrared spectroscopy. As shown in Fig. 4, the peaks at 3300, 3250, 1602, 1298, 750, 495, and 476 cm^−1^ were the characteristic of *cis*-DIDP (Fig. 4a). Figure 4b shows that the PCL nanofibers were amorphous. From the spectra, the retention of amorphous PCL nanofibers was observed in the structure of *cis*-DIDP@PCL (Fig. 4c) with the peaks of *cis*-DIDP.

### Release Profile In Vitro and Drug Loading Efficiency

**Fig. 5:**
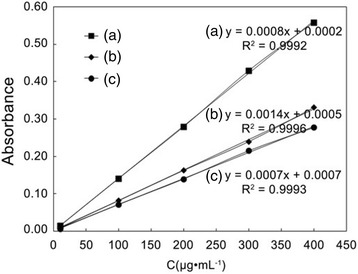
Curves of UV absorbance to concentration of *cis*-DIDP in **a** deionized water, **b** normal saline, and **c** PBS

The *cis-*DIDP concentration in solution was determined by UV–Vis spectrophotometer. The absorption of *cis-*DIDP at 298 nm in solution (deionized water, normal saline, or PBS) was observed to be proportional to the concentration (Fig. 5). The linear regression was respectively expressed in the following equations.

2 $$ y = 0.0008 x + 0.0002 $$

**Fig. 6:**
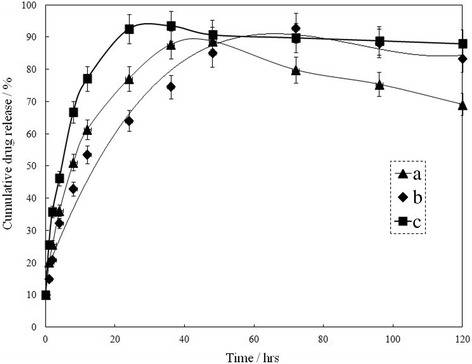
Release profiles of *cis*-DIDP from the *cis*-DIDP@PCL fibers in **a** deionized water, **b** normal saline, and **c** PBS, at room temperature. Each data point represents the average of three times

In Eq. (2), the correlation coefficient is 0.9992 (Fig. 6a).

3 $$ y = 0.0014 x + 0.0005 $$

In Eq. (3), the correlation coefficient is 0.9996 (Fig. 6b).

4 $$ y = 0.0007 x + 0.0007 $$

In Eq. (4), the correlation coefficient is 0.9993 (Fig. 6c).

In these equations, *y* is the absorption and *x* is the concentration of *cis-*DIDP. Based on these equations, the amount of the *cis-*DIDP was measured over time and the release profiles of *cis-*DIDP from *cis*-DIDP@PCL were obtained in different solutions. The cumulative concentration of *cis-*DIDP released from *cis*-DIDP@PCL in different solution was calculated by Eqs. (2)–(4).

Figure 6 shows the release profiles of *cis-*DIDP from *cis-*DIDP@PCL (*cis-*DIDP, PCL = 1:10) in (a) deionized water, (b) normal saline, and (c) PBS. The release rate of *cis-*DIDP was faster in normal saline than that in deionized water, but it was a little slower in PBS. When the drug accumulative release reached the maximum, there was a trend that the curve declines at different degrees. This phenomenon could be further confirmed that the hydrolysis of *cis-*DIDP occurs in solution as discussed above, but *cis-*DIDP did not hydrolyze completely. The PCL nanofibers were dispersed in deionized water, and the PCL polymers were uniformly distributed in solution, which inhibited the *cis-*DIDP hydrolysis. The serious burst release did not appear in the initial release of *cis-*DIDP from *cis-*DIDP@PCL, indicating that *cis-*DIDP was better incorporated into nanofibers. The concentration of *cis-*DIDP was observed to reach its maximum earlier in normal saline (about 24 h) than that in deionized water (about 48 h), and in PBS (about 72 h). Then, the concentration of *cis-*DIDP decreased gradually, and the downward trend was most obvious in deionized water, moderate in PBS, and weakest in normal saline. The results indicated that the presence of Cl^−^ promoted the release of *cis-*DIDP from *cis-*DIDP@PCL but inhibited its hydrolysis. As shown in Fig. 6c, the controlled release of *cis-*DIDP from *cis-*DIDP@PCL might be gained for long term in PBS. *Cis-*DIDP@PCL (100 mg) was dissolved in 100 mL deionized water. The concentration of free *cis-*DIDP in the solution was measured by UV–Vis spectroscopy for three times. Because of uniform dispersion of *cis-*DIDP in electrospinning solution and scattering in products, the encapsulation efficiency of product could be calculated to be 88.87% (EE%, Eq. (1)).

### Scheme of Electrospinning and Sustained-Release Process

**Scheme 1:**
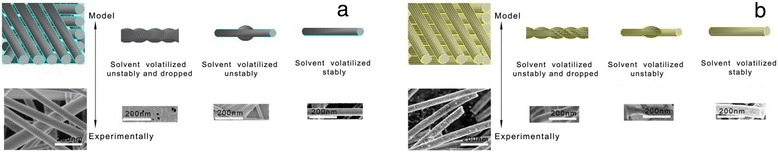
Blank PCL fibers and *cis*-DIDP@PCL fibers were electrospun under different conditions

According to the results discussed above, we outlined the schemes of electrospinning solution preparation and sustained-release process. As shown in Additional file 1: Scheme S1, PCL powders were added into DMF by stirring to form PCL mucus as the blank PCL electrospinning solution. *Cis-*DIDP was dispersed in DMF, then dispersed in blank PCL electrospinning solution to form PCL containing *cis-*DIDP electrospinning solution. Then, the solution was respectively electrospun to obtain blank PCL nanofibers and *cis*-DIDP@PCL (Scheme 1).

The model process of *cis-*DIDP sustained-release from *cis-*DIDP@PCL in solution was exhibited in Additional file 1: Scheme S2. At the beginning, *cis-*DIDP soon dropped from the *cis*-DIDP@PCL surfaces and dispersed into the solution. The initial concentration of *cis-*DIDP was approximately 10%. As time goes on, *cis-*DIDP continuously released from *cis*-DIDP@PCL and the concentration of *cis-*DIDP increased gradually. Finally, the *cis-*DIDP released almost completely and uniformly dispersed with extremely slow hydrolysis in solution, while PCL nanofibers formed a layer of film.

### Anticancer Activity In Vitro

**Fig. 7:**
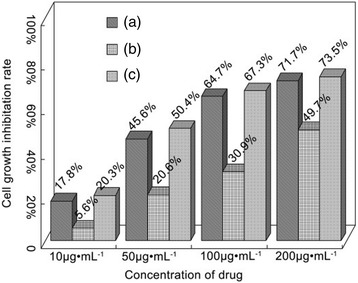
Anticancer activities to human hepatocellular carcinoma cell SMMC-7721 line cell in vitro. **a** Cisplatin. **b** *Cis*-DIDP. **c** *Cis*-DIDP@PCL

The anticancer activity of *cis-*DIDP@PCL against human hepatocellular carcinoma cells (SMMC-7721 line cell) was investigated with MTT assay. The *cis-*DIDP@PCL was directly added to the tumor-cell-cultured well and incubated for 24 h. The anticancer activity of free cisplatin and *cis-*DIDP was tested as controls. Seen from Fig. 7, in the cases of actual *cis-*DIDP content 10, 50, 100, and 200 μg mL^−1^ in the nanofibers, the cell growth inhibition rates of 20.3, 50.4, 67.3, and 73.5% are achieved and are a little better than that of free cisplatin, for the rates of 17.8, 45.6, 64.7, and 71.7%, respectively, and much better than free *cis*-DIDP at rates of 5.6, 20.6, 30.90, and 49.7%, respectively. That is to say, the same amount of drug from free cisplatin and *cis-*DIDP@PCL are almost of equal anticancer activity in vitro. However, the anticancer activity of free *cis-*DIDP is much lower for its hydrolysis. The IC_50_ value (concentration of drug able to inhibit the growth of SMMC-7721 line cells to 50% of the control) of the free cisplatin, free *cis-*DIDP, and *cis-*DIDP released from nanofibers had been determined. The result showed that the IC_50_ value was ~60 μg/mL (free cisplatin), ~200 μg/mL (free *cis-*DIDP), and ~50 μg/mL (*cis-*DIDP released from PCL nanofibers), respectively. The results showed that *cis-*DIDP became sustained-release from the PCL nanofibers in solution and preserved the better inhibition effect. The *cis-*DIDP@PCL was a sustained drug vehicle, and *cis-*DIDP could be continuously released from the systems. Therefore, the shortcomings of *cis-*DIDP, such as instability and poor solubility in the human body, can be overcome. Incorporating *cis-*DIDP into the nanofibers by electrospun should be an ideal technique for improving the performance of the *cis-*DIDP.

## Conclusions

According to the molecular structure analysis, the anticancer activity of *cis*-DIDP was better than that of cisplatin. However, there was little research on the clinical application of *cis*-DIDP for its unstability, which defect is overcome by incorporating *cis*-DIDP into the carriers. Meanwhile, the common toxicity and cross-resistance of platinum-based anticancer drugs have also been inhibited. In this work, the controlled-release systems of *cis-*DIDP from the electrospun carriers were tested, in which c*is-*DIDP was finely incorporated into the PCL nanofibers. It is obviously effective that *cis-*DIDP sustained-releases from the nanofibers inhibit human lung tumor cells in vitro. The results show that *cis-*DIDP@PCL are ideal controlled-release drug carrier, and the vehicle may be applied in clinic. It is instructive to improve other inorganic anticancer drug anticancer chemotherapy with the same method. The total evaluation of the system in vivo would be confirmed after more perfect evaluation in vitro, and the results would be further verified by animal experiments. If achieved good results, there would be potential for clinical trials.

## Additional file

Additional file 1: Figure S1. Structure of complexes: (a) cisplatin; (b) *Cis*-DIDP. **Figure S2.** UV absorbance changes with time of 1 mmol L^−1^ *cis*-DIDP (a) and cisplatin in 0.9% saline. □-0 h, ◇-6 h, △-12 h, ×-24 h, ○-48 h, *-96 h. **Figure S3.** UV absorbance changes with time of 1 mmol L^−1^ *cis*-DIDP (a) and cisplatin (b) in 5% glucose. □-0 h, ◇-6 h, △-12 h, ×-24 h, ○-48 h, *-96 h. **Figure S4.** UV absorbance changes with time of 1 mmol L^−1^ *cis*-DIDP (a) and cisplatin in 0.1 mol L^−1^ glycine. □-0 h, ◇-6 h, △-12 h, ×-24 h, ○-48 h, *-96 h. **Table S1.** Average diameters and morphology of fibers shown in Additional file 1: Figure S5 under different ratio of PCL/*cis*-DIDP. **Figure S5.** SEM micrographs of fibers fabricated by different ratio of PCL to *cis*-DIDP: (a) 100/0, (b) 100/10, (c) 100/100, and (d) 100/150. **Table S2.** Average diameters and morphology of fibers shown in Additional file 1: Figure S6 under different voltage. **Figure S6.** SEM micrographs of fibers fabricated by different voltage: (a) 10, (b) 15, (c) 20, and (d) 25 kV. **Table S3.** Average diameters and morphology of fibers shown in Additional file 1: Figure S7 under different distance. **Figure S7.** SEM micrographs of fibers fabricated by different distance: (a) 10, (b) 15, (c) 20, and (d) 25 cm. **Table S4.** Average diameters and morphology of fibers shown in Additional file 1: Figure S8 under different flow rate. **Figure S8.** SEM micrographs of fibers fabricated by different flow rates: (a) 0.5, (b) 1.0, (c) 2.0, and (d) 3.0 mL h^−1^. **Scheme S1.** Preparation of electrospinning solution. **Scheme S2.** *Cis*-DIDP@PCL sustained-release model with time. (DOC 4296 kb)
