# Supplementary material for: Electrospun Poly(ε-caprolactone) Composite Nanofibers with Controlled Release of Cis-Diamminediiodoplatinum for a Higher Anticancer Activity
Source: Nanoscale Res Lett. 2017 Apr 28;12:318. doi: 10.1186/s11671-017-2092-y (PMC5408359; doi:10.1186/s11671-017-2092-y)
Supplement: Additional file 1: Figure S1. — Structure of complexes: (a) cisplatin; (b) Cis-DIDP. Figure S2. UV absorbance changes with time of 1 mmol L−1 cis-DIDP (a) and cisplatin in 0.9% saline. □-0 h, ◇-6 h, △-12 h, ×-24 h, ○-48 h, *-96 h. Figure S3. UV absorbance changes with time of 1 mmol L−1 cis-DIDP (a) and cisplatin (b) in 5% glucose. □-0 h, ◇-6 h, △-12 h, ×-24 h, ○-48 h, *-96 h. Figure S4. UV absorbance changes with time of 1 mmol L−1 cis-DIDP (a) and cisplatin in 0.1 mol L−1 glycine. □-0 h, ◇-6 h, △-12 h, ×-24 h, ○-48 h, *-96 h. Table S1. Average diameters and morphology of fibers shown in Additional file 1: Figure S5 under different ratio of PCL/cis-DIDP. Figure S5. SEM micrographs of fibers fabricated by different ratio of PCL to cis-DIDP: (a) 100/0, (b) 100/10, (c) 100/100, and (d) 100/150. Table S2. Average diameters and morphology of fibers shown in Additional file 1: Figure S6 under different voltage. Figure S6. SEM micrographs of fibers fabricated by different voltage: (a) 10, (b) 15, (c) 20, and (d) 25 kV. Table S3. Average diameters and morphology of fibers shown in Additional file 1: Figure S7 under different distance. Figure S7. SEM micrographs of fibers fabricated by different distance: (a) 10, (b) 15, (c) 20, and (d) 25 cm. Table S4. Average diameters and morphology of fibers shown in Additional file 1: Figure S8 under different flow rate. Figure S8. SEM micrographs of fibers fabricated by different flow rates: (a) 0.5, (b) 1.0, (c) 2.0, and (d) 3.0 mL h−1. Scheme S1. Preparation of electrospinning solution. Scheme S2. Cis-DIDP@PCL sustained-release model with time. (DOC 4296 kb) [file 11671_2017_2092_MOESM1_ESM.doc]

**Electrospun Poly(ε-caprolactone) Composite Nanofibers with Controlled Release of *cis*-Diamminediiodoplatinum for a Higher Anticancer Activity**

Chaojing Mu, and Qingsheng Wu*

School of Chemical Science and Engineering, Shanghai Key Lab of Chemical Assessment and Sustainability, Tongji University, Shanghai, 200092, China

* **Corresponding author:** Tel: +86 21 6598 2620, Fax: +86 21 6598 1097

E-mail: [qswu@tongji.edu.cn](mailto:qswu@tongji.edu.cn), [02121@tongji.edu.cn](mailto:or02121@tongji.edu.cn)


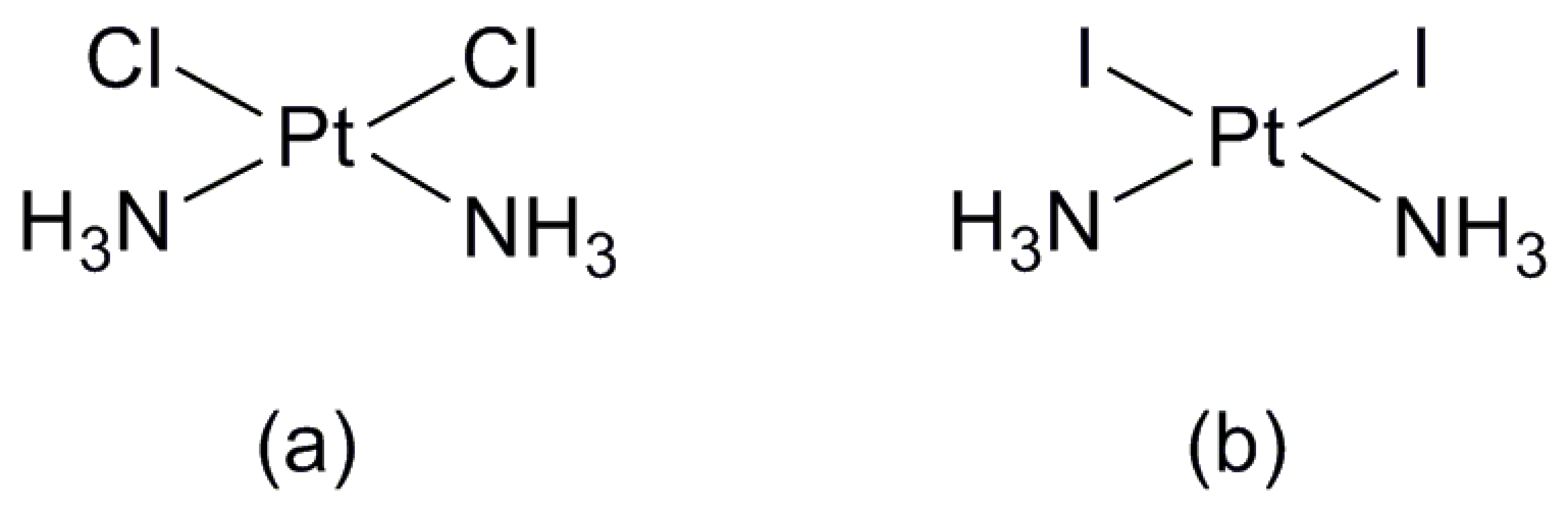


**Additional file 1: Figure S1**. Structure of complexes: (a) Cisplatin; (b) *Cis*-DIDP.


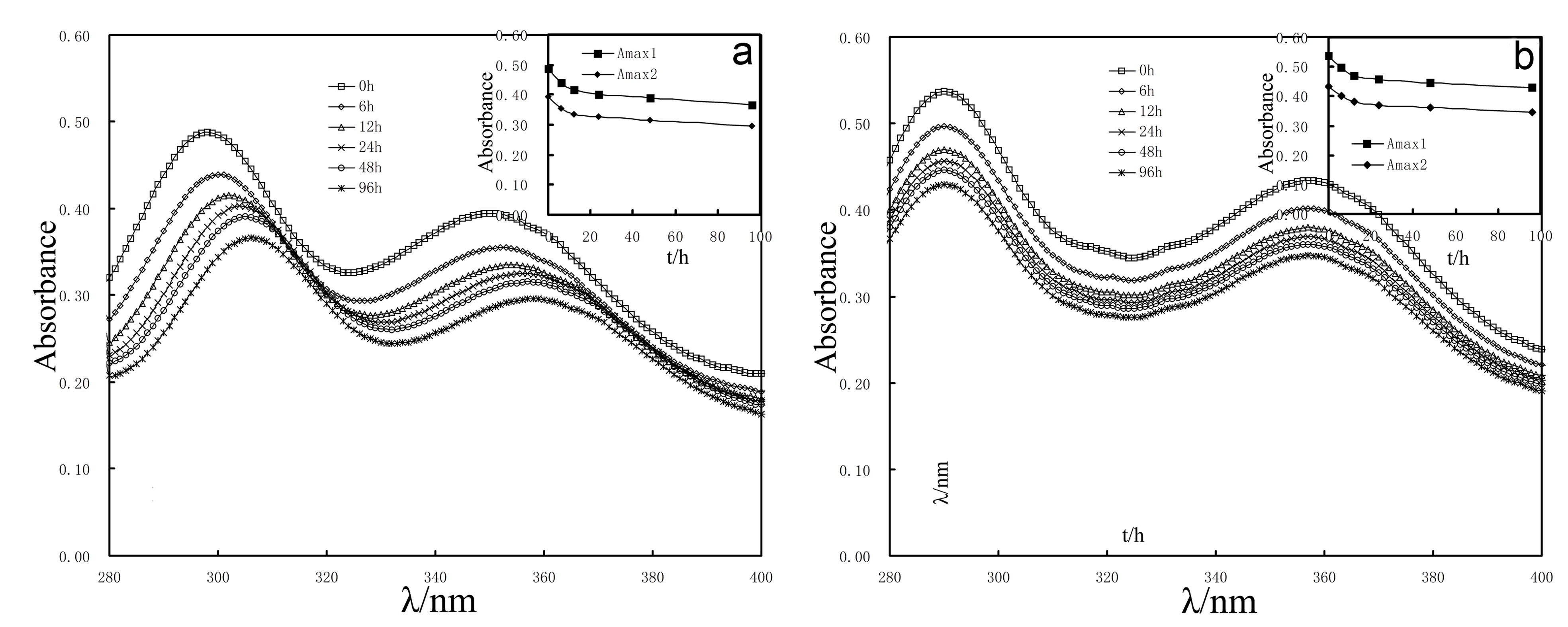


**Additional file 1: Figure S2**. UV absorbance changes with time of 1mmol·L^-1^ *cis*-DIDP (a) and cisplatin in 0.9%saline. □-0h, ◇-6h, △-12h, ×-24h, ○-48h, *-96h.


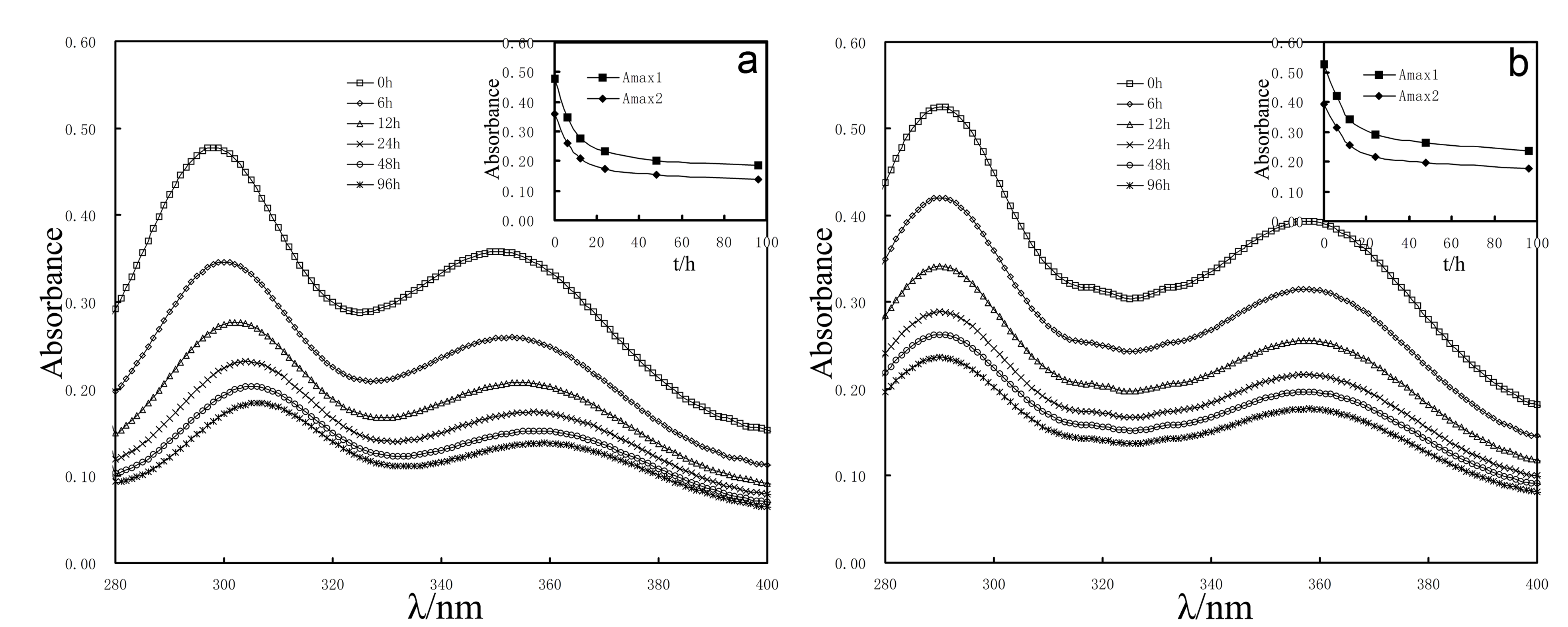


**Additional file 1: Figure S3**. UV absorbance changes with time of 1mmol·L^-1^ *cis*-DIDP (a) and cisplatin (b) in 5%glucose. □-0h, ◇-6h, △-12h, ×-24h, ○-48h, *-96h.


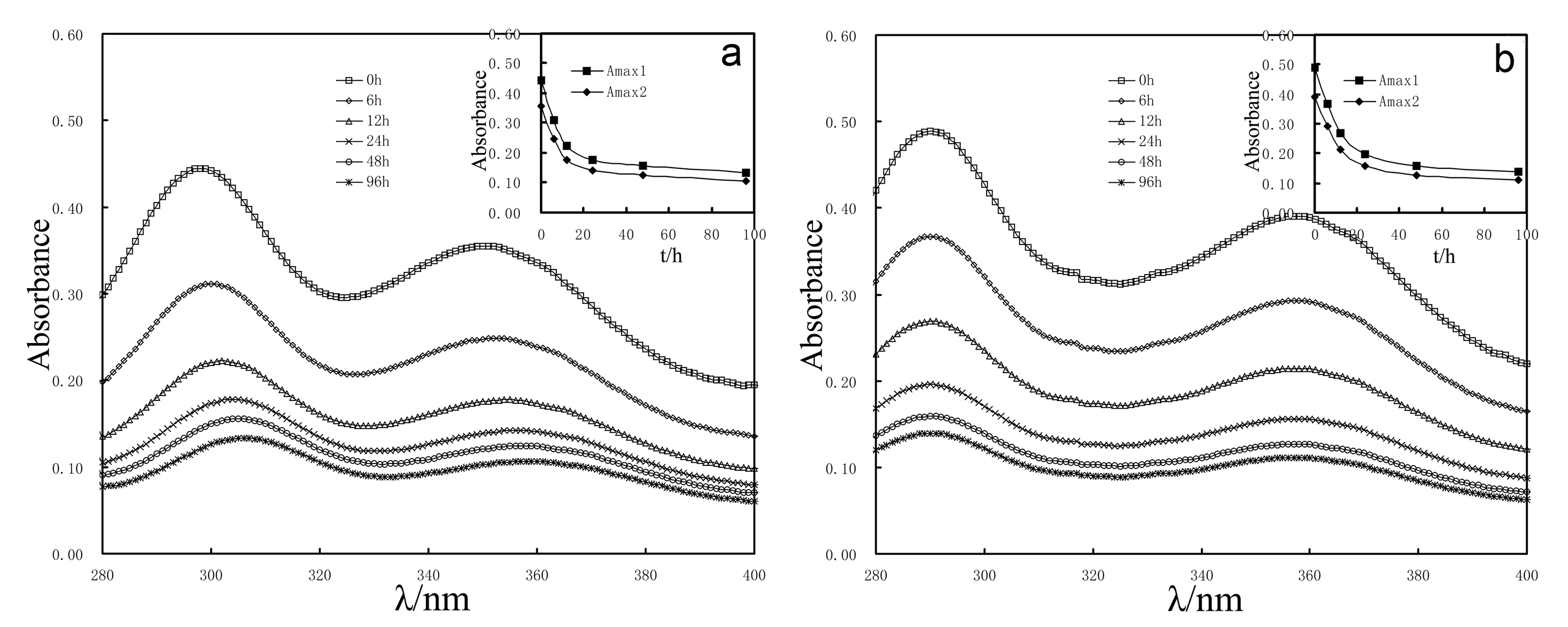


**Additional file 1: Figure S4**. UV absorbance changes with time of 1mmol·L^-1^ *cis*-DIDP (a) and cisplatin in 0.1 mol·L^-1^ glycine. □-0h, ◇-6h, △-12h, ×-24h, ○-48h, *-96h.

**Fabrication conditions of the products.** The fibers average diameters from 50 to 500 nm could be finely tuned by adjusting the electrospinning processing parameters, such as *cis-*DIDP concentration, solvent, electrospinning voltage, polymer solution flow rates, the distances between needle and collector, etc. Figure S5-S8 respectively shows the SEM images of the products fabricated under different conditions. Different operation parameters are respectively shown in Table S1-S4. SEM morphology refers respectively to the fibers fabricated by adjusting the mass fraction of *cis-*DIDP to PCL (FigureS5), the electrospinning voltages (Figure S6), the distances between needle and collector (Figure S7), or the polymer solution flow rates (Figure S8). The fabricating conditions and fibers average diameters are separately shown in Table S1 (different *cis-*DIDP amount), Table S2 (different voltages), Table S3 (different distances), and Table S4 (different flow rates). In this work, we found that when the proportion of the *cis-*DIDP/PCL was more than 15% (FigureS5d), or the voltages lower than 15 kV (FigureS6a, b), or the distances shorter than 10 cm (FigureS7a) and longer than 20 cm (FigureS7d), or the flow rates larger than 2.0 mL·h^-1^ (Figure S8d), fibers contained a few droplets. Higher voltages (higher than 25 kV, Figure S6d) led equipment malfunction interrupt the electrospinning process. Lower flow rates (lower than 0.5 mL·h^-1^) might block the needle. When the *cis-*DIDP/PCL was more than 15%, the fiber was mainly instead by a large number of droplets. So it is important to select appropriate range of electrospinning conditions. 10/100 (*cis-*DIDP/PCL), 20 kV (voltage), 1.0 mL·h^-1^ (flow rate), 15 cm (distance) were selected as the operating parameters to fabricate products in the following studies.

**Additional file 1: Table S1**. Average diameters and morphology of fibers shown in Figure S5 under different ratio of PCL/*cis*-DIDP.

| Sample | Ratio of PCL /*cis*-DIDP | Voltage | Flow rate | Distance | Diameters | Morphology |
| --- | --- | --- | --- | --- | --- | --- |
| a | 1.0g/0mg | 20KV | 1.0mL·h^-1^ | 15cm | 50-200nm | Fiber |
| b | 1.0g/10mg | 20KV | 1.0mL·h^-1^ | 15cm | 50-200nm | Fiber |
| c | 1.0g/100mg | 20KV | 1.0mL·h^-1^ | 15cm | 100-150nm | Fiber |
| d | 1.0g/150mg | 20KV | 1.0mL·h^-1^ | 15cm | - | Seriously bonded |


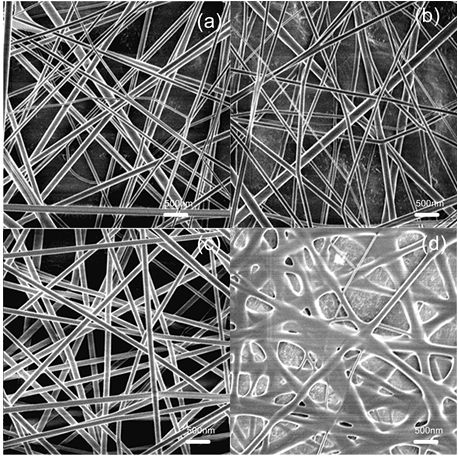


**Additional file 1: Figure S5**. SEM micrographs of fibers fabricated by different ratio of PCL to *cis*-DIDP: (a) 100/0, (b) 100/10, (c) 100/100, (d) 100/150.

**Additional file 1: Table S2**. Average diameters and morphology of fibers shown in Figure S6 under different voltage.

| Sample | Ratio of PCL /*cis*-DIDP | Voltage | Flow rate | Distance | Diameters | Morphology |
| --- | --- | --- | --- | --- | --- | --- |
| a | 1.0g/100mg | 10KV | 1.0mL·h-1 | 15cm | - | Seriously dropped |
| b | 1.0g/100mg | 15KV | 1.0mL·h-1 | 15cm | 100-500nm | Bonded |
| c | 1.0g/100mg | 20KV | 1.0mL·h-1 | 15cm | 100-150nm | Fiber |
| d | 1.0g/100mg | 25KV | 1.0mL·h-1 | 15cm | 100-500nm | Distorted |


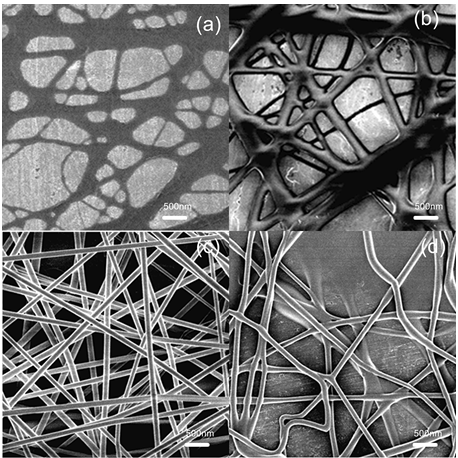


**Additional file 1: Figure S6**. SEM micrographs of fibers fabricated by different voltage: (a) 10, (b) 15, (c) 20, and (d) 25 kV.

**Additional file 1: Table S3**. Average diameters and morphology of fibers shown in Figure S7 under different distance.

| Sample | Ratio of PCL /*cis*-DIDP | Voltage | Flow rate | Distance | Diameters | Morphology |
| --- | --- | --- | --- | --- | --- | --- |
| a | 1.0g/100mg | 20KV | 1.0mL·h-1 | 10cm | - | Droplet |
| b | 1.0g/100mg | 20KV | 1.0mL·h-1 | 15cm | 100-150nm | Fiber |
| c | 1.0g/100mg | 20KV | 1.0mL·h-1 | 20cm | 100-500nm | Bonded |
| d | 1.0g/100mg | 20KV | 1.0mL·h-1 | 25cm | - | Seriously bonded |


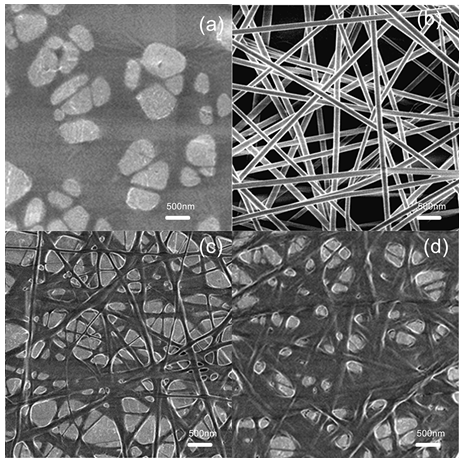


**Additional file 1: Figure S7**. SEM micrographs of fibers fabricated by different distance: (a) 10, (b) 15, (c) 20, and (d) 25 cm.

**Additional file 1: Table S4**. Average diameters and morphology of fibers shown in Figure S8 under different flow rate.

| Sample | Ratio of PCL /*cis*-DIDP | Voltage | Flow rate | Distance | Diameters | Morphology |
| --- | --- | --- | --- | --- | --- | --- |
| a | 1.0g/100mg | 20KV | 0.5 mL·h-1 | 15cm | 50-400nm | Fiber |
| b | 1.0g/100mg | 20KV | 1.0mL·h-1 | 15cm | 100-150nm | Fiber |
| c | 1.0g/100mg | 20KV | 2.0mL·h-1 | 15cm | 50-400nm | Fiber |
| d | 1.0g/100mg | 20KV | 3.0mL·h-1 | 15cm | - | Seriously bonded |


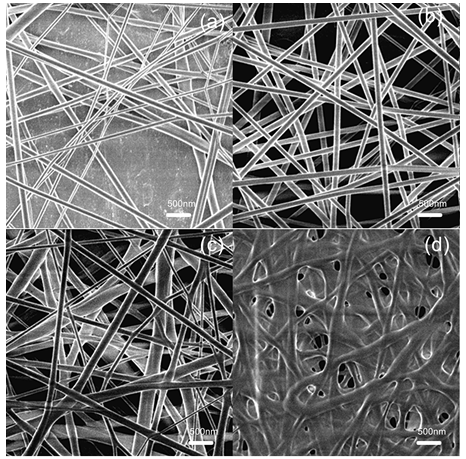


**Additional file 1: Figure S8**. SEM micrographs of fibers fabricated by different flow rate: (a) 0.5, (b) 1.0, (c) 2.0, and (d) 3.0 mL·h^-1^.


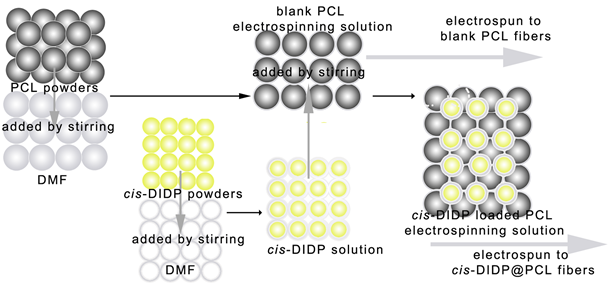


**Additional file 1: Scheme S1.** Preparation of electrospinning solution.


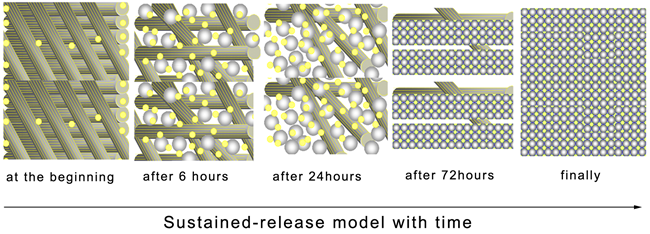


**Additional file 1: Scheme S2.** *Cis*-DIDP@PCL sustained-release model with time.
